# SGLT2 Inhibitors and Liver Cirrhosis: Hype or Hope?

**DOI:** 10.3390/life15121788

**Published:** 2025-11-21

**Authors:** Olga Brusnic, Danusia Maria Onisor, Adrian Boicean, Corina Porr, Florin Daniel Sofonea, Paula Anderco, Cristian Ichim

**Affiliations:** 1 Department of Internal Medicine VII, George Emil Palade University of Medicine, Pharmacy, Science and Technology of Targu Mures, Gheorghe Marinescu Street No. 38, 540136 Targu Mures, Romania; brusnic_olga@yahoo.com (O.B.);; 2 Faculty of Medicine, Lucian Blaga University of Sibiu, 550169 Sibiu, Romania; adrian.boicean@ulbsibiu.ro (A.B.); corina_sibiu@yahoo.com (C.P.); cristian.ichim@ulbsibiu.ro (C.I.); 3 Faculty of Social Sciences, Lucian Blaga University of Sibiu, 550012 Sibiu, Romania

**Keywords:** SGLT2i, liver cirrhosis, ascites

## Abstract

Liver cirrhosis is marked by sodium and water retention, portal hypertension and sharply reduced survival after decompensation. Sodium–glucose cotransporter-2 inhibitors (SGLT2i) induce insulin-independent glycosuria and natriuresis and have proven cardio-renal benefits, prompting interest in their role as adjuncts for ascites. This review synthesizes current evidence on efficacy, safety and mechanistic plausibility of SGLT2i in cirrhosis. Observational cohorts and case series suggest that adding SGLT2i to standard diuretics increases natriuresis, lowers ascites burden and paracentesis requirements, improves weight and aminotransferases and may reduce hepatic decompensation and hepatocellular carcinoma risk. Safety remains paramount: hypotension, acute kidney injury and hepatorenal syndrome-related acute kidney injury, genitourinary infections, electrolyte disturbances and rare euglycemic ketoacidosis necessitate careful patient selection, slow titration and close monitoring, especially in decompensated disease and when combined with loop diuretics or mineralocorticoid receptor antagonists. Overall, the balance of data supports cautious optimism: SGLT2i represent a promising adjunct within protocolized care pathways for selected patients, while definitive trials powered for hepatic outcomes are still required to clarify indications, timing, dosing and long-term impact.

## 1. Introduction

Advanced chronic liver disease ranks sixteenth among contributors to the global disease burden, accounting for 1.8% of disability-adjusted life years in 2019 [1]. Worldwide deaths from cirrhosis rose from roughly 676,000 in 1980 to more than 1 million across 128 countries by 2010 [2]. Decompensation, signaled by complications of portal hypertension or hepatocellular dysfunction, includes variceal hemorrhage, ascites, jaundice and hepatic encephalopathy, each of which substantially diminishes survival and quality of life [3]. Ascites is the most common first decompensating event, occurring in approximately half of patients within a decade, and its onset carries an estimated 20% annual mortality [4,5].

Management of cirrhosis-related ascites relies on dietary sodium restriction and diuretics, chiefly loop agents and mineralocorticoid receptor antagonists, with refractory cases necessitating procedures such as large-volume paracentesis, transjugular intrahepatic portosystemic shunt or ultimately liver transplantation [6,7]. Survival declines markedly once the disease progresses to the decompensated stage [8]. Prognosis remains unfavorable: patients with compensated cirrhosis live about 12 years on average, but survival drops to around 2 years once decompensation develops [3]. To date, no established therapy reliably halts or reverses the progression of hepatic fibrosis and its sequelae [9].

Sodium–glucose cotransporter-2 (SGLT2) is expressed in the kidney’s proximal tubule, where it mediates reabsorption of glomerularly filtered glucose [10]. It accounts for approximately 90–95% of total renal glucose reabsorption [11]. Loss-of-function variants in SGLT2 impair this process and present clinically as familial renal glucosuria (“renal diabetes”) [12]. SGLT2 inhibitors (SGLT2i) are antihyperglycemic agents that work by blocking the SGLT2 cotransporter in the kidney’s proximal convoluted tubule, thereby interrupting tubular reuptake of filtered glucose and sodium [13]. This mechanism is distinct because it functions independently of pancreatic β-cell activity or insulin sensitivity [14]. It promotes glycosuria and natriuresis, yielding an osmotic diuresis with higher urinary glucose excretion and increased urine output [15,16].

Beyond glucose lowering, large trials have shown broad cardiovascular protection, most notably fewer cardiovascular deaths and fewer hospitalizations for heart failure, effects thought to stem in part from their natriuretic/diuretic action and the resultant attenuation of renin–angiotensin–aldosterone system (RAAS) activation [17,18]. By dampening RAAS tone, these drugs improve hemodynamic stability and reduce myocardial wall stress, which underpins their inclusion in contemporary guidelines for heart failure across the ejection-fraction spectrum, irrespective of diabetes status [19].

Empagliflozin was shown to exhibit additional therapeutic benefits, including uricosuric and natriuretic effects, as well as well-documented nephroprotective and cardioprotective actions [20]. Moreover, it exerts pleiotropic effects through multiple pathways, such as activation of the AMPK–autophagy axis and demonstrates antioxidant activity [21]. Studies have also shown that empagliflozin can improve hepatic steatosis and fibrosis in patients with non-alcoholic fatty liver disease, even in the absence of diabetes [22]. It modulates SIRT-1 in cardiac tissue and hypoxia-inducible factors in renal tissue; however, its specific actions within hepatic tissue remain to be elucidated [23,24].

Since the early-2024 review on SGLT2i use in cirrhosis, the field has expanded, including publication of the first two randomized controlled trials evaluating these agents for refractory ascites across forms of liver cirrhosis [25,26,27]. This article aims to present the most up-to-date evidence on the effects of SGLT2i in liver cirrhosis.

## 2. Biology and Pharmacology of SGLT2 and Its Inhibitors

SGLTs are membrane transport proteins that actively reabsorb sodium-coupled carbohydrates against their concentration gradients [28,29]. The family comprises six members that preferentially mediate monosaccharide uptake [30]. SGLT1 transports glucose and galactose, SGLT2 transports glucose, SGLT3 functions primarily as a glucose sensor, SGLT4 and SGLT5 recognize fructose and mannose and SGLT6 participates in myo-inositol absorption [31]. Among these, SGLT1 and SGLT2 are the best studied and are the targets of recently developed inhibitory therapies [30]. SGLT transporters bind sodium and monosaccharides at a shared binding site [31]. Consequently, SGLT1 couples transport of one monosaccharide to one sodium ion, whereas SGLT2 couples glucose transport to two sodium ions [32].

The kidney is central to the actions of agents that modify blood pressure, glycaemia and body weight [33]. This is due in part to SGLT2, located in the S1 segment of the proximal convoluted tubule, which helps maintain glucose homeostasis [33]. Under normal physiology, roughly 180 g of glucose are filtered each day, about 90% of which is reabsorbed via SGLT2 together with approximately 65% of filtered sodium [34]. When plasma glucose exceeds ~180 mg/dL, the renal threshold for glucose reabsorption, glycosuria ensues [34]. In type 2 diabetes mellitus, the maximal renal capacity for glucose reabsorption is increased, likely through SGLT2 upregulation, though the literature is not uniform on this point [35,36]. Enhanced proximal reabsorption of glucose and sodium reduces sodium delivery to the macula densa, perturbs tubule-glomerular feedback, induces afferent arteriolar vasodilation and elevates intraglomerular pressure [37].

SGLT2i offer cardiorenal protection in both diabetic and non-diabetic populations [38,39]. Benefits arise from coordinated metabolic effects: blocking proximal tubular glucose reabsorption to promote glycosuria and hemodynamic actions, including natriuresis with blood-pressure lowering [40,41]. Additional mechanisms include improvements in renal and vascular function: modulation of energy metabolism and endothelial signaling pathways, a shift in substrate utilization toward lipids and ketone bodies and attenuation of oxidative stress [42,43,44]. Collectively, these pleiotropic effects highlight heart–kidney biochemical cross-talk and are associated with protection across diverse cardiovascular and renal conditions. In diabetes, chronic hyperglycaemia drives impaired insulin secretion, insulin resistance, glucotoxicity and oxidative stress, thereby elevating cardiovascular and renal risk [45,46,47]. Notably, SGLT2 inhibition mitigates tubular glucotoxicity by reducing glucose reabsorption, alleviates mitochondrial dysfunction and renal hypoxia by lowering oxygen demand, and may support β-cell function [16,48,49].

Four SGLT2i (canagliflozin, dapagliflozin, empagliflozin and ertugliflozin) are FDA-approved as add-ons to diet and exercise to enhance glycemic control in adults with type 2 diabetes [50]. Drawing on prior outcome trials and their study populations, canagliflozin and empagliflozin also carry indications to lower cardiovascular death risk in adults with type 2 diabetes and established cardiovascular disease [51,52].

Although a robust body of work supports SGLT2i use in earlier stages of metabolic dysfunction, associated steatotic liver disease (MASLD), with reported reductions in liver stiffness, hepatic enzymes and visceral adiposity, along with improved insulin sensitivity and a potential slowdown in progression to cirrhosis, evidence in advanced disease remains limited [50,53,54,55,56]. The same glycosuric action that underpins their antidiabetic efficacy also increases susceptibility to genitourinary infections, a concern that may be magnified in cirrhosis owing to impaired immune defenses [57,58]. Moreover, as with loop diuretics, the baseline circulatory instability of many patients with cirrhosis, characterized by hypotension and RAAS dysregulation, can heighten the risk of acute kidney injury and/or hepatorenal syndrome, disturb electrolyte balance, exacerbate hepatic encephalopathy and potentially contribute to sarcopenia, which adversely affects long-term outcomes [59,60,61,62,63].

Large retrospective database analyses suggest that SGLT2i therapy may be associated with lower mortality and fewer hepatic decompensation events in cirrhosis, with signals of benefit in both compensated and decompensated settings [7,59,64,65]. Complementing these observations, case reports, case series and multiple systematic and narrative reviews describe scenarios where SGLT2, despite possible adverse effects, function as useful adjuncts for refractory ascites when combined with standard diuretics (loop agents and mineralocorticoid receptor antagonists) [25,66,67,68,69]. More recently, experimental studies and additional case series have expanded this emerging evidence base, focusing on SGLT2i for fluid management in cirrhosis [26,27,70,71,72].

Major adverse effects of SGLT2i include genital mycotic infections (including Fournier’s gangrene), urinary tract infections (UTI) (including urosepsis and pyelonephritis), increased risk of limb amputation, euglycaemic diabetic ketoacidosis, dyslipidaemia, volume depletion leading to acute kidney injury (AKI) and hypokalaemia [73]. These risks are particularly concerning in cirrhosis, especially in decompensated disease, where severe hepatic dysfunction manifests as ascites, variceal hemorrhage, hepatic encephalopathy, hepatorenal syndrome (HRS) and jaundice [74]. In decompensation, portal hypertension drives splanchnic vasodilation and systemic hypotension, reducing renal perfusion [75].

Loop diuretics can precipitate volume depletion and hypotension, provoking AKI or HRS-AKI, and a similar risk may arise from the natriuretic effect of SGLT2i when combined with standard-of-care diuretics [76]. This combination can also exacerbate sodium and potassium disturbances, to which cirrhotic patients are already predisposed [77]. SGLT2i increases rates of genital mycotic infection and may raise UTI risk at higher doses, a particular concern in cirrhosis with impaired innate immunity [78,79,80]. They are also associated with diabetic ketoacidosis, sometimes with normal glucose (euglycaemic diabetic ketoacidosis) in type 2 diabetes mellitus [81,82]. Patients with cirrhosis experience worse in-hospital outcomes during diabetic ketoacidosis, and SGLT2i–triggered diabetic ketoacidosis has been reported in alcoholic cirrhosis, where active alcohol use and starvation further promote ketoacidosis [83,84,85].

Empagliflozin was studied across Child–Pugh A–C with a single 50 mg dose, and although exposure rose modestly with advancing hepatic impairment, urinary glucose excretion and the overall pharmacodynamic profile were preserved, and routine dose adjustment was not recommended, while cirrhosis-specific safety data remain limited and warrant careful monitoring [86]. Feasibility work in advanced chronic liver disease further showed the expected glucosuric and natriuretic effects with acceptable short-term tolerability, supporting careful, protocolized use in research settings and emphasizing the need for surveillance in decompensated states [6].

## 3. Pathophysiology of Liver Cirrhosis and Its Complications

Liver cirrhosis and its complications remain an unresolved clinical challenge. Although direct-acting antivirals have lowered hepatitis C prevalence, cirrhosis attributable to alcohol use and nonalcoholic steatohepatitis continue to increase [87].

Sodium and water retention in cirrhosis reflects extra-renal mechanisms rather than an intrinsic kidney defect, as kidneys from end-stage liver disease donors function normally after transplant into recipients with preserved hepatic function. Ascites formation is multifactorial [88]. Portal hypertension arises from increased intrahepatic resistance to portal inflow caused by distortion of vascular architecture [89]. Sinusoidal cell changes drive vasoconstriction, while perisinusoidal chronic inflammatory infiltrates and activation of hepatic stellate cells further narrow the sinusoids through cytokine signaling and direct intercellular interactions [90]. Vasoregulatory homeostasis is profoundly disturbed: the splanchnic bed is dilated and less responsive to vasoconstrictors and multiple vasoactive mediators are dysregulated [88,91].

Ascites in cirrhosis is classically explained by two paradigms: overflow and underfilling [88,91]. In the overflow framework, heightened intrahepatic vascular resistance with elevated sinusoidal pressure provokes renal sodium retention independent of volume status [88,92]. Liver fibrosis augments hepatic afferent nerve traffic and triggers an adenosine-mediated hepatorenal reflex; notably, A1-receptor blockade prevents sodium retention in cirrhotic rat models, supporting this reflex as an early driver of ascites [88]. Volume-independent sodium retention can then expand plasma volume, increase pressure across the portosplanchnic circuit, and generate “overflow” ascites; with progression, accumulating fluid may compress the renal vein and produce congestive renal dysfunction/failure [93,94].

By contrast, the underfilling model posits that increased hepatic resistance plus hypoalbuminaemia fosters peritoneal fluid transudation, leading to effective hypovolaemia [95]. Because plasma volume expansion requires adequate oncotic pressure, overflow physiology typically predominates in earlier cirrhosis when serum albumin is relatively preserved, whereas underfilling becomes more apparent with disease progression [95,96,97]. Additional contributors to circulatory underfilling include peripheral vasodilation with an attenuated vasoconstrictor response, arteriovenous shunting, cirrhotic cardiomyopathy, occult gastrointestinal bleeding, and volume depletion from excessive diuretic use [98]. Vasodilation initially affects the splanchnic circulation and later becomes systemic, culminating in arterial underfilling [99].

In cirrhosis, vascular dysregulation reflects the combined action of vasoconstrictive/antinatriuretic and vasodilatory/natriuretic pathways [100]. Major constrictors encompass endothelin, eicosanoids, the RAAS, arginine vasopressin (antidiuretic hormone) and sympathetic outflow, whereas nitric oxide, glucagon, carbon monoxide, prostacyclin and endocannabinoids predominate among dilators [101,102]. As a compensatory reaction to effective underfilling, vasoconstrictor systems, especially the RAAS, are engaged early, with wound-healing-type local signaling and elevated circulating angiotensin II documented in cirrhosis [103].

Although RAAS blockade with ACE inhibitors or angiotensin II type 1 receptor antagonists reduces hepatic fibrogenesis in preclinical and clinical contexts, these agents are contraindicated in decompensated cirrhosis due to hypotension and risk of hepatorenal syndrome; however, they may have value in slowing progression during earlier disease stages [104,105,106]. Nitric oxide plays a central role across multiple cirrhotic phenotypes: hyperdynamic circulation, sodium/water retention, hepatopulmonary syndrome and cirrhotic cardiomyopathy, while systemic arterial vasodilation diminishes effective arterial blood volume, precipitating renal functional decline and hyponatraemia [107].

Portal hypertension is a hallmark complication of cirrhosis and the principal driver of hepatic decompensation [108]. Structural remodeling, fibrosis and parenchymal nodularity as fixed (mechanical) factors, together with sinusoidal endothelial dysfunction as a dynamic component, initiate an increase in intrahepatic vascular resistance and elevate portal pressure [108,109]. Subsequent splanchnic arterial vasodilation induces systemic cardiovascular adaptations that culminate in a hyperkinetic state with high cardiac output, reduced systemic vascular resistance, and fluid retention [110,111]. In the setting of persistently high hepatic resistance, the increased splanchnic inflow further amplifies portal pressure [112]. At this stage, patients develop clinically significant portal hypertension, wherein the risk of a first decompensating event rises proportionally with portal pressure [113,114].

## 4. Therapeutic Role of SGLT2i in Cirrhosis

This class appears to benefit ascites that is unresponsive to maximal doses of standard-of-care diuretics and may also help when those agents are contraindicated due to adverse effects, mirroring observations in heart failure, where they can enhance diuresis and permit reductions in loop diuretic dosing [115]. In a pilot study of refractory ascites, the agents tripled 24 h urinary sodium at one month, with the effect sustained at three months alongside a reduction in excess water retention [71]. A recent case series previewing an ongoing trial in decompensated cirrhosis reported substantial decreases in ascites grade in all participants, with complete resolution at six months in most cases, despite initiation primarily for cardiovascular risk rather than ascites [70].

**Figure 1:**
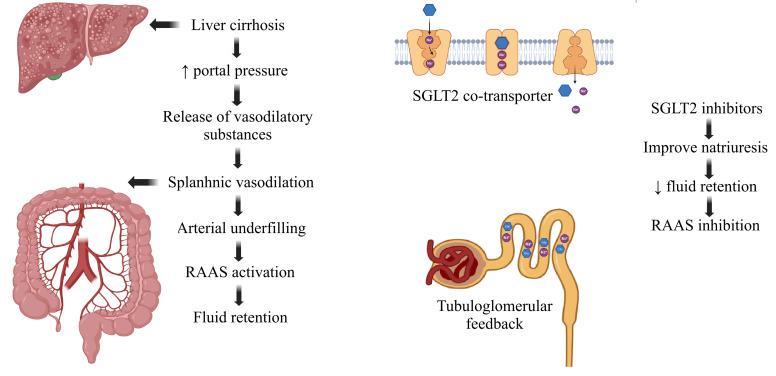
SGLT2i effects in liver cirrhosis.

Hyponatraemia frequently accompanies refractory ascites in decompensated cirrhosis, and its severity portends worse outcomes, including when serum sodium is only mildly reduced (130–135 mEq/L) [116,117]. These agents have been proposed as a means to improve hyponatraemia, with case-level observations showing correction of low sodium after initiation in decompensated cirrhosis, including a rise from 133 mEq/L to normonatraemic levels despite ongoing natriuresis [53,69,118]. By contrast, in patients who are normonatraemic at baseline, serum sodium generally does not increase with SGLT2i therapy, consistent with reports in type 2 diabetes where canagliflozin and dapagliflozin produced only modest or non-significant changes in serum sodium [69,119,120,121]. Figure 1 summarizes the pathophysiologic cascade from portal hypertension to RAAS-driven sodium–water retention and highlights where SGLT2 inhibition intervenes.

A mechanistic explanation is provided by SGLT2i–induced osmotic diuresis: enhanced urinary glucose raises tubular fluid osmolarity, limiting tubular sodium and water reabsorption and promoting excretion of both sodium and sodium-independent water, which can stabilize or even increase serum sodium despite natriuresis [122,123,124]. Additional hormonal adaptations may contribute: reductions in atrial natriuretic peptide have been observed under SGLT2 blockade in experimental heart failure and in clinical diabetes, potentially tempering natriuresis, while rises in serum sodium and osmolality can stimulate arginine vasopressin release, with its secretion during treatment correlating positively with urinary glucose and sodium excretion [125,126].

**Figure 2:**
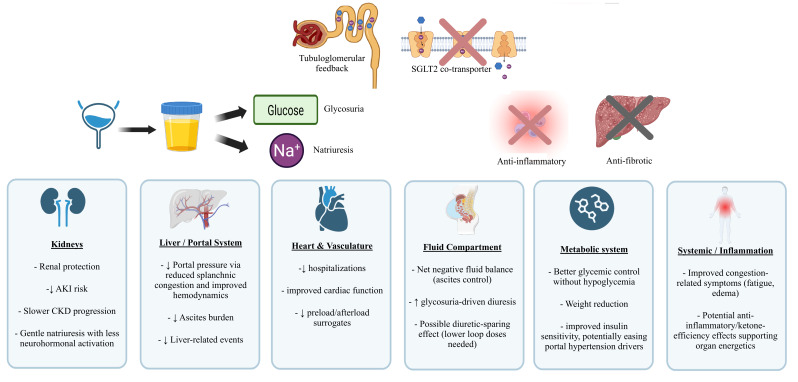
SGLT2i effects in liver cirrhosis—mechanisms and potential benefits.

Fluid-compartment data using bioimpedance suggest the drugs preferentially reduce extracellular (particularly interstitial) water when pre-treatment Extracellular Water/Total Body Water is high, with minimal change when it is low, and may remove proportionally more interstitial than intravascular fluid compared with other diuretics, features that could help reduce ascites while limiting dehydration and intravascular volume loss [120,127]. Overall, in cirrhotic patients with hyponatraemia, SGLT2i can induce natriuresis alongside sodium-independent water excretion and neurohumoral adjustments that together tend to raise, rather than lower, serum sodium [122,123,124]. However, dedicated studies are needed to define efficacy, safety and patient selection for sodium regulation in this population. Figure 2 presents, at the organ level, the proposed effects of SGLT2i in cirrhosis, including reduced portal pressure and ascites, renal protection and cardiometabolic benefits.

Early interventional work, including a prospective feasibility study in diuretic-resistant ascites and a randomized trial in refractory ascites, suggests that add-on SGLT2 inhibition can facilitate ascites control and reduce the need for large-volume paracentesis over short horizons, provided careful selection and monitoring are applied [27,128]. In clinical practice, a prudent approach is to consider initiation in patients within Child–Pugh B or at the lower end of B/C, when MELD-Na and bedside indicators point to hemodynamic stability, namely preserved mean arterial pressure, absence of active infection or AKI/HRS-AKI and at least moderate renal function while avoiding initiation during acute decompensation or sepsis [98,129,130]. This framing aligns with contemporary guidance for decompensated cirrhosis, which integrates portal-hypertensive burden, sodium disorders, and renal trajectory into therapeutic decision-making [129].

Reductions in hepatic inflammation, reflected by lower ferritin and aminotransferase levels and plausibly mediated by decreased oxidative stress, may translate into antifibrotic effects that lower liver stiffness and, in turn, portal hypertension, the key driver of variceal bleeding [55,56,74,131,132,133]. In patients with severe hepatic fibrosis (liver stiffness > 10 kPa), SGLT2i use was associated with reductions in liver stiffness on vibration-controlled transient elastography [131]. Although cirrhosis was not confirmed in all participants, these patients resembled compensated cirrhosis in MASLD, where liver stiffness > 12.0 kPa is typical [134]. Accordingly, the inhibitors may ameliorate fibrosis in early cirrhosis and, thus, have a potential role in compensated disease, as suggested by a recent review [25]. A cohort study further supports this, reporting a lower incidence of esophageal varices, lesions closely linked to portal hypertension and liver stiffness, among users of these agents [132].

Experimental work shows that SGLT2i slow architectural changes driving portal hypertension in carbon tetrachloride–induced cirrhosis in rats and clinical data indicate a reduction in clinically significant portal hypertension when combined with zibotentan in compensated cirrhosis of diverse etiologies [133,135]. Mendelian randomization using genetic proxy inhibition of SGLT2 has also been associated with lower hazards of hepatic decompensation, implying potential improvement in liver function with this class [73,136]. In compensated cirrhosis, observational data, predominantly in MASLD, showed mean weight loss of about 3.4 kg and reductions in transaminases, consistent with attenuated hepatic inflammation and adiposity similar to findings in non-cirrhotic MASLD [55,56,72]. Another cohort analysis further noted significant decreases in paracentesis frequency at one, three, and six months after treatment initiation [73].

Episodes of hepatic encephalopathy may also decline through reductions in portal pressure and decreased reliance on loop diuretics, which can lessen hypokalaemia, a known precipitant of encephalopathy [74]. Longer-term hepatic benefits are suggested by a meta-analysis associating SGLT2i exposure with lower hepatocellular carcinoma incidence in older adults with T2DM and/or MASLD, alongside reduced risks of other gastrointestinal cancers [137]. In decompensated cirrhosis, compilations of case reports and series describe this therapy as a useful adjunct for refractory ascites, acting synergistically with standard-of-care diuretics: loop diuretics and mineralocorticoid receptor antagonists [53,67,138].

Potential harm must be weighed carefully in cirrhosis. The natriuretic effect can aggravate pre-existing hypotension and prospective data in compensated disease show meaningful declines in mean arterial pressure over three months [72,139]. These haemodynamic patterns align with heart-failure evidence, particularly in heart failure with preserved ejection fraction, where these drugs improved patient-reported outcomes and exercise capacity and reduced heart-failure hospitalizations irrespective of diabetes status [140,141,142]. Excessive diuresis may also reduce renal perfusion and trigger AKI or HRS-AKI in cirrhosis, both linked to high mortality [143]. While electrolyte disturbances are common in cirrhosis, most case series and prospective reports with this therapy describe mild increases or normalization of serum sodium [26,27,77]. Rarely, euglycaemic diabetic ketoacidosis has been observed shortly after initiation of the class in alcoholic cirrhosis with ongoing alcohol use, underscoring the need for vigilance in this subgroup [84].

Cirrhotic patients exhibit immunosuppression due to impaired synthesis of antibacterial proteins across both innate and adaptive pathways, predisposing them to bacterial infections [57]. Because this therapy induces glycosuria, it has been hypothesized that it elevates the risk of bacterial UTI [58]. Combined with the underlying immune dysfunction of cirrhosis, this would suggest a higher UTI risk among cirrhotic patients receiving the class. However, in our cohort, we observed only a trend toward increased bacterial UTI in those with decompensated cirrhosis. This observation is consistent with data from the general population using these inhibitors [144]. Notably, a subgroup analysis within a meta-analysis reported a significant rise in UTI with treatment durations exceeding one year [79].

Cirrhotic cardiomyopathy is an infrequent, typically late complication of cirrhosis, for which liver transplantation remains the only definitive option beyond mineralocorticoid receptor antagonists and β-blockers [145]. Both diastolic and systolic dysfunction can develop as the disease advances [145]. It is characterized by elevated cardiac output, blunted contractile responsiveness to stress, and impaired diastolic relaxation, and may be present in up to half of individuals with cirrhosis [146,147]. Tissue Doppler imaging studies indicate that subclinical systolic dysfunction can be detected at rest, not solely during increased metabolic demand [148]. Early longitudinal strain abnormalities resemble the pattern observed in diabetic cardiomyopathy [149,150,151]. In trials of SGLT2i, treatment has been associated with improved longitudinal strain and reductions in indexed left ventricular mass over six months, suggesting potential cardioprotective effects relevant to this phenotype [25,152]. Although these pathophysiological parallels are compelling, clinical evidence in cirrhotic cardiomyopathy is lacking, with only a single case report noting improved systemic vascular resistance and haemodynamics after starting the drug in decompensated cirrhosis [68].

Table 1 consolidates clinical, translational and mechanistic evidence indicating that SGLT2i may aid ascites control and favorably modulate portal and hepatic pathophysiology in cirrhosis, while outlining safety considerations in decompensated disease.

## 5. Biomarkers and Molecular Targets for Response Prediction

Identifying patients most likely to benefit from SGLT2 inhibition may be facilitated by pragmatic pharmacodynamic and volume-status biomarkers adapted from diabetes, chronic kidney disease and decongestion studies, pending validation in cirrhosis [163,164].

The spot urinary glucose-to-creatinine ratio (uGCR) is an accessible readout of on-treatment glycosuria and has been proposed as a screening tool to estimate SGLT2i effect size and to flag patients with unexpectedly low urinary glucose excretion [165]. In CREDENCE adjunct analyses, greater glycosuria under canagliflozin aligned with stronger protection from cardio-renal outcomes, supporting uGCR or related glycosuria metrics as a candidate response biomarker [166].

Across cardiovascular outcome data, on-treatment rises in hematocrit and hemoglobin were the strongest mediators of the reduction in cardiovascular death with empagliflozin, supporting these as practical response markers [167,168]. This hematologic signal reflects both hemoconcentration and an SGLT2i-induced activation of erythropoiesis evidenced by early increases in erythropoietin and erythroferrone with coordinated suppression of hepcidin [169,170]. Accordingly, hemoglobin/hematocrit trajectories may function as low-burden pharmacodynamic biomarkers of effective decongestion under SGLT2 inhibition, while acknowledging that liver-specific validation is pending [171].

Renal albuminuria reduction has emerged as a treatment effect marker under SGLT2 inhibitors, aligning with improved kidney outcomes across trials. Although SGLT2i confer renal benefit even when albuminuria changes are modest, on-therapy decreases in albuminuria remain a clinically meaningful indicator of response worth prospective testing in cirrhosis cohorts [172].

SGLT2 inhibitors consistently lower serum uric acid and urate dynamics have been proposed as a mechanistic and prognostic biomarker of treatment effect [173]. Network and systematic meta-analyses confirm class-wide urate lowering, supporting serum uric acid change as a feasible, inexpensive marker to monitor pharmacologic response [174].

For congestion-linked phenotypes, natriuretic peptides (NT-proBNP) track hemodynamic improvement under SGLT2 inhibition in heart failure, although effect sizes and patterns vary by disease severity and setting [175,176]. Model-based analyses further indicate that baseline NT-proBNP and renal function modify the magnitude of NT-proBNP decline during SGLT2i therapy, suggesting a role in stratifying expected benefit [177].

Because water handling is central to decompensation, copeptin, a stable vasopressin surrogate, represents a liver-disease–specific biomarker that correlates with circulatory dysfunction and prognosis in cirrhosis and could be explored to anticipate water-balance responses to SGLT2 inhibition [178]. Recent data reinforce copeptin’s prognostic value in decompensated advanced chronic liver disease, supporting its candidacy for risk stratification and monitoring [179].

## 6. Risks and Uncertainties

Although converging signals suggest benefit, several uncertainties warrant a balanced appraisal of SGLT2i in cirrhosis [18]. The durability of decongestion is debated, with physiological work indicating that early reductions in extracellular water may be counter-regulated over time, attenuating sustained natriuresis [159]. In comparative analyses outside cirrhosis, SGLT2i induce a distinct decongestive profile that can be smaller than loop-diuretic–driven shifts at usual clinical doses, underscoring heterogeneity in fluid responses [180]. Conversely, bioimpedance-guided data suggest preferential interstitial vs. intravascular fluid removal during SGLT2i therapy, mechanistically attractive for ascites control with less effective hypovolemia, yet these findings remain to be validated specifically in cirrhosis [180]. Early interventional studies in cirrhosis (pilot and randomized) indicate improved ascites control, but follow-up intervals are short and liver-specific outcomes require longer trials [27,71].

Infectious risk needs also nuance: randomized and observational syntheses consistently show a clear increase in genital mycotic infections, while the association with UTI is mixed overall and may be more evident with higher doses or longer exposure [181,182]. Given immune dysfunction in cirrhosis, any incremental genitourinary risk could carry disproportionate clinical consequences, reinforcing careful selection and monitoring [181]. Because sarcopenia worsens prognosis in cirrhosis, the pattern seen with SGLT2 inhibition, preferential fat-mass loss accompanied by small but measurable lean-mass reductions, argues for nutrition-aware initiation with longitudinal tracking of weight, strength, and electrolytes in this population [183,184].

Euglycemic ketoacidosis, uncommon but clinically significant, has been increasingly recognized with SGLT2i and may be precipitated by starvation, acute illness or alcohol use, all relevant in advanced liver disease [185,186]. High clinical suspicion, patient education, and prompt discontinuation are essential when compatible symptoms occur [185].

## 7. Conclusions

SGLT2i offer a biologically coherent and clinically promising adjunct to standard care for cirrhosis, particularly in patients with ascites, by coupling insulin-independent glycosuria with natriuresis, partial restoration of tubuloglomerular feedback and preferential interstitial fluid mobilization. Across early randomized data, observational cohorts and case-level reports, signals converge on improved ascites control, fewer procedure needs, modest weight and enzyme improvements and possible benefits on liver stiffness and portal hemodynamics. These advantages align with the class’s established cardio-renal effects and suggest a role in carefully selected cirrhotic populations.

Safety is the fulcrum. Hypotension, AKI/HRS-AKI, genitourinary infections, electrolyte shifts and rare euglycemic ketoacidosis mandate protocolized initiation (low dose, slow uptitration), baseline and serial monitoring of blood pressure, renal function and electrolytes and heightened vigilance in decompensated disease or when combined with loop diuretics/mineralocorticoid antagonists. Alcohol use disorder, advanced sarcopenia, and recent infections warrant particular caution.

On balance, the evidence supports cautious hope rather than hype: SGLT2i should be considered as adjuncts within structured pathways, ideally embedded in prospective programs with predefined stop rules and rescue options.

## Figures and Tables

**Table 1:** Evidence on SGLT2i in cirrhosis and advanced liver disease (Abbreviations: SGLT2i: sodium–glucose cotransporter-2 inhibitor, ACLD: advanced chronic liver disease, MASLD: metabolic dysfunction-associated steatotic liver disease, T2DM: type 2 diabetes mellitus, SoC: standard of care (loop + mineralocorticoid receptor antagonist), PK/PD: pharmacokinetics/pharmacodynamics, AE: adverse event, HCC: hepatocellular carcinoma, CKD: chronic kidney disease, RAAS/SNS: renin–angiotensin–aldosterone system/sympathetic nervous system, ER stress: endoplasmic reticulum stress, CCl_4_: carbon tetrachloride).

Study Type/Model	Population/Liver Context	SGLT2i	Key Findings	Safety	Ref
**A. Preclinical/Translational**					
Preclinical (CCl_4_ cirrhotic rat)	Experimental cirrhosis with portal hypertension	Tofogliflozin	Lower portal pressure/intrahepatic resistance; antifibrotic, anti-angiogenic, endothelial-supportive effects	No renal toxicity signal reported in study	[133]
Ex vivo vascular reactivity	Mesenteric arteries (hypertensive vs. control)	Empagliflozin (acute)	Direct vasorelaxation via SIRT1/AMPK-linked pathways; plausible splanchnic benefit	—	[153]
Preclinical (toxic injury/fibrosis)	Liver injury/fibrosis model	Class (e.g., empagliflozin)	Reduced oxidative/ER stress; antifibrotic and histologic improvement	—	[9]
Preclinical (hepatotoxicity)	Liver injury (non-cirrhotic)	Dapagliflozin ± hepatoprotective co-therapy	Enzyme and histologic improvements; antioxidant pathway activation	—	[154]
Mendelian randomization + mouse	Fibrosis risk (human MR) and NAFLD model (mouse)	Empagliflozin (mouse arm)	Genetic proxies linked to lower fibrosis/cirrhosis risk; reduced steatosis/fibrotic markers in mice	—	[155]
Mechanistic (HFD-MASLD mouse)	Early metabolic liver disease	Empagliflozin	Less steatosis; improved oxidative-stress signaling (NRF1 axis)	—	[156]
**B. Clinical–Observational ( cirrhosis /MASLD and related)**					
Retrospective cohort (propensity-matched)	Cirrhosis with T2DM (metformin users)	Class (add-on to metformin)	Association with fewer hepatic decompensation events and lower HCC risk	Observational; class-typical infections noted in background literature	[64]
Real-world cohort (propensity-matched)	MASLD (predominantly non-cirrhotic at baseline)	Class	Association with slower progression to advanced liver disease and better long-term survival	Observational signals; metabolic improvements consistent with class	[157]
Two case reports (longitudinal)	Decompensated cirrhosis with ascites and T2DM	Luseogliflozin	Clinically meaningful, durable ascites control; recurrence off-drug and improvement on re-challenge	No major AEs reported across extended follow-up in cases	[158]
**C. Clinical–Interventional/PK–PD**					
Open-label PK/PD and safety	ACLD (compensated and decompensated)	Empagliflozin	Expected glucosuric/natriuretic pharmacodynamics; supports feasibility in ACLD	Short-term safety acceptable; AE profile broadly class-consistent	[6]
Randomized controlled trial	Decompensated cirrhosis, refractory ascites (SoC vs. SoC + empagliflozin)	Empagliflozin	Add-on improved ascites control and reduced procedure need in the short term	Mostly mild AEs (e.g., cramps, hyponatraemia) overall acceptable in study window	[27]
Prospective comparative cohort (mechanistic decongestion)	CKD (non-liver)–fluid compartment analysis	Dapagliflozin ± conventional diuretics	Combo reduced interstitial/extracellular water with relative plasma-volume preservation (mechanistic relevance to ascites)	No excess RAAS/SNS activation with combination in study	[159]
Prospective head-to-head (non-cirrhotic)	MASLD with T2DM	Dapagliflozin vs. oral semaglutide	Both improved metabolic/liver measures; contextual support for liver-stiffness benefits earlier in disease	Expected class safety; no specific liver safety signal	[160]
Randomized, double-blind (non-liver; vascular)	Systemic hemodynamics in T2DM	Empagliflozin vs. dapagliflozin	Faster improvement in arterial stiffness (hemodynamic relevance to hyperdynamic circulation)	—	[161]
**D. Reviews/Perspectives (context and trial design)**					
Narrative/clinical perspective	Cirrhosis and ascites management context	Class	Summarizes emerging clinical signals for refractory ascites and outlines trial considerations	Emphasizes patient selection and monitoring in decompensation	[162]

## Data Availability

No new data were created or analyzed in this study.
